# The potentials of carbon monoxide-releasing molecules in cancer treatment: An outlook from ROS biology and medicine

**DOI:** 10.1016/j.redox.2021.102124

**Published:** 2021-09-06

**Authors:** Thi Thuy Tien Vo, Quang Canh Vo, Vo Phuoc Tuan, Yinshen Wee, Hsin-Chung Cheng, I-Ta Lee

**Affiliations:** a School of Dentistry, College of Oral Medicine, Taipei Medical University, Taipei, Taiwan; b Department of Dental Biomaterials Science, Dental Research Institute and BK21 Plus Program, School of Dentistry, Seoul National University, Seoul 03080, Republic of Korea; c Endoscopy Department, Cho Ray Hospital, Ho Chi Minh City, Viet Nam; d Department of Pathology, University of Utah, Salt Lake City, UT, USA; e Department of Dentistry, Taipei Medical University Hospital, Taipei, Taiwan

**Keywords:** Carbon monoxide, Carbon monoxide-releasing molecules, Reactive oxygen species, Pro-tumorigenic pathways, Apoptosis, Warburg effect

## Abstract

Carbon monoxide (CO) is now well recognized a pivotal endogenous signaling molecule in mammalian lives. The proof-of-concept employing chemical carriers of exogenous CO as prodrugs for CO release, also known as CO-releasing molecules (CO-RMs), has been appreciated. The major advantage of CO-RMs is that they are able to deliver CO to the target sites in a controlled manner. There is an increasing body of experimental studies suggesting the therapeutic potentials of CO and CO-RMs in different cancer models. This review firstly presents a short but crucial view concerning the characteristics of CO and CO-RMs. Then, the anticancer activities of CO-RMs that target many cancer hallmarks, mainly proliferation, apoptosis, angiogenesis, and invasion and metastasis, are discussed. However, their anticancer activities are varying and cell-type specific. The aerobic metabolism of molecular oxygen inevitably generates various oxygen-containing reactive metabolites termed reactive oxygen species (ROS) which play important roles in both physiology and pathophysiology. Although ROS act as a double-edged sword in cancer, both sides of which may potentially have been exploited for therapeutic benefits. The main focus of the present review is thus to identify the possible signaling network by which CO-RMs can exert their anticancer actions, where ROS play the central role. Another important issue concerning the potential effect of CO-RMs on the aerobic glycolysis (the Warburg effect) which is a feature of cancer metabolic reprogramming is given before the conclusion with future prospects on the challenges of developing CO-RMs into clinically pharmaceutical candidates in cancer therapy.

## Highlights

• CO-RMs as pro-drugs for controlled CO delivery are potentially beneficial in cancer treatment.
• Anticancer activities of CO-RMs are varying and cell-type specific.
• Anti-proliferative, pro-apoptotic, and anti-angiogenic effects are major niches.
• ROS may play a central role in the molecular pathways underlying anticancer activities of CO-RMs.
• CO-RMs can act against Warburg effect, a feature of cancer metabolic reprogramming.

## Graphical abstract

**Figure:**
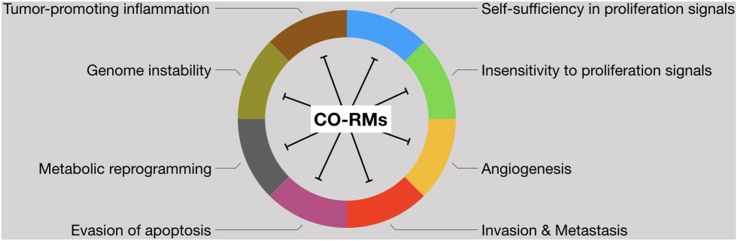

## 1 Introduction

Cancer is typically a generic term that confers a group of diseases characterized by the uncontrolled cell growth and the acquisition of metastasis [1]. Based on the GLOBOCAN 2020 data, there is an estimation of over 19 million new cancer cases and around 10 million cancer deaths worldwide in 2020, resulting in a leading cause of morbidity and mortality all over the world. It is also predicted that the global burden of cancer would increase by 47% from 19.3 million new cases in 2020 to 28.4 million cases in 2040, and this increase in the incidence would be accompanied by the increase in the mortality rates [2]. Clearly, cancer has yielded a significant issue for public health. For the sake of human wellness, the research and development of feasible, effective, and affordable strategies for cancer treatment is one of priority areas in the fight against cancer.

The universal concept of oxidative stress is referred to as “an imbalance between oxidants and antioxidants in the favor of the oxidants, leading to a disruption of redox signaling and control and/or molecular damage” [3]. Despite of remaining ambiguous, the discovery of the involvement of oxidative stress during the course of cancer can be considered a noticeable milestone in cancer biology. A series of reviews has reported that the disturbance of redox balance may result in numerous oncogenic cellular events, such as genome instability and mutations, uncontrolled cell proliferation, evasion of cell death, angiogenesis, invasiveness, and metastasis, among others [[4], [5], [6]]. On the other hand, chronic inflammation has also been shown to involve in multiple steps in carcinogenesis [7,8]. Moreover, it has been documented that oxidative stress can trigger chronic inflammation through the activation of various transcription factors that modulate the expression of a wide variety of growth factors, cytokines, chemokines, cell cycle regulatory molecules, so forth [5,9]. Interestingly, while oxidative stress can lead to inflammation, the opposite that is inflammation can induce oxidative stress may also occur [10]. Therefore, oxidative stress and inflammation coordinately involve in a self-perpetuating cycle that can foster cancer. From this perspective, the application of therapeutic agents targeting both processes may be beneficial in cancer treatment.

Since the first introduction of carbon monoxide (CO) in the literature over a century ago, CO has been long regarded as a silent killer as a result of hypoxia secondary to CO occupation of oxygen binding sites on hemoglobin [11]. The lethal reputation of CO, however, has been drastically changed owing to the fact that it is endogenously produced in the body through the breakdown of heme via heme oxygenase (HO) enzymes [[12], [13], [14], [15], [16], [17]]. To date, CO is widely accepted not simply a deleterious gas but an important molecule for cellular signaling in mammalian systems [11]. During recent years, a multitude of experimental models and preclinical settings have shown the benefits of the enhancement of endogenous CO production and the delivery of exogenous CO, indicating promising therapeutic potentials of CO in many diseases, including cancer [18,19]. Boosting endogenous CO generation through the induction of HO represents a complicated scenario, and it is beyond the scope of this review. Although the pure form of gaseous CO has been reported to exert beneficial effects in different experimental settings, the clinical translation of CO administration via inhalation remains challenging mainly due to tissue unspecificity and safety issues [20]. Consequently, there has been great endeavor for crafting an array of compounds that spatially and temporally liberate CO into biological systems in a controlled manner, also known as CO-releasing molecules (CO-RMs), providing a promising alternative to the inhalation of gaseous CO in clinical practice [[21], [22], [23], [24], [25]].

CO-RMs were initially implemented to mimic the function of the stress inducible isoform of HO that degrades heme into CO and biliverdin to yield anti-oxidant and anti-inflammatory actions [[21], [22], [23]]. So far, CO-RMs have been experimentally demonstrated to elicit similar effects as compared to gaseous CO, particularly anti-inflammatory activities [24,25]. Considering the linkage between oxidative stress and inflammation with cancer, these compounds have captured the interest of researchers as potential anticancer candidates. In fact, a recent review has presented a comprehensive overview of therapeutic potentials of CO-RMs in different types of cancer, including breast cancer, prostate cancer, colon cancer, cervical cancer, pancreatic cancer, skin cancer, lung adenocarcinoma, lymphoma, and acute myeloid leukemia [26]. Furthermore, CO-RMs have been also reported to prevent adverse side effects caused by other anticancer modalities, such as doxorubicin-induced cardiotoxicity, cisplatin-induced nephrotoxicity, and radiation-induced secondary cancer, suggesting additional benefits of these compounds in cancer therapy [26]. Albeit CO-RMs have been proposed to be implicated in cancer treatment, the relevant research is only at an early stage with contentious results. Importantly, still little is known about the cellular events and molecular pathways regulated by CO liberated from these donors in the context of cancer. Therefore, this review attempts to shed light on the cytoprotective effects and molecular mechanisms of CO in the form of CO-RMs underlying the anticancer action of these CO-based therapeutic compounds. Herein, the main theme is the analysis of possible signaling network which may provide more insights on the anticancer activities of CO derived from CO-RMs according to the redox perspective.

## 2 Carbon monoxide: from killer to healer

CO has been long regarded as a gaseous pollutant in the atmosphere which originates from the incomplete combustion of organic matter [27]. Discovered in the late 18th century, CO was originally labelled as a toxic molecule [28]. Living beings typically encounter CO at varying levels through inhalation of contaminated air [27], and incidental CO poisoning remains an issue of concern worldwide [29]. Following a series of experiments indicating the interplay among oxygen, CO, and hemoglobin (Hb), Drs. Douglas, Haldane and Haldane first proposed the carboxyhemoglobin (COHb) theory of CO poisoning. The scholars found that CO is able to bind to Hb to form COHb, and the affinity of CO for Hb is about 300 times greater than that of oxygen [30]. Therefore, CO can compete oxygen for binding to Hb, leading to the displacement of oxygen which in turn reduces oxygen carrying capacity and oxygen off-loading capacity. As a result of the disruption of blood oxygen transportation, the toxicity of CO is primarily attributed to tissue hypoxia, and injury is secondary to hypoxia [11]. The severity of CO poisoning varies according to the exposure concentration and duration of CO which can be reflected by the COHb level. Mild symptoms can manifest between 10 and 20% COHb, and death can occur at COHb greater than 60% [31]. However, the histopathology and clinical course of CO poisoning is yet properly advocated by the COHb theory only. Now we have discovered more sophisticated targets of CO referring to as “extra-hemoglobin effects”, [11,32]. Of significance, mitochondria which contain heme-based cytochromes can also be the target of high concentrations of CO, compromising the cellular respiratory function [[33], [34], [35]]. Therefore, it is necessary to aware that CO poisoning is a complicated process that primarily involves hypoxia but not entirely.

Intriguingly, the evidence that CO is present in the body dates back to 1894 when Gréhant described the presence of a combustible gas in blood [12], which was later suspected to be CO [13]. However, it was impossible to assert the origin of CO in the blood with available methods in those days. It was not until 1949 that the endogenous production of CO was discovered [15]. Twenty years later, the source of endogenous CO was ascertained to be the enzyme system capable of degrading heme so-called heme oxygenase (HO) [16,17]. Since then, we have been witnessing the explosion in the quantity and quality of studies of the actions of CO in mammalian systems. More than 75% of CO produced in human body arises from the erythrocyte turnover [36], while a small fraction (20%) originates from other hemoproteins such as myoglobin and iron-containing enzymes [31]. CO is generally produced at very low levels during the metabolism in healthy adults that result in the background COHb concentration in blood of approximately 0.5–0.8% [37]. CO was initially considered as a by-product of heme degradation without purposeful physiological function. To date, CO has been recognized to function as an important signaling molecule more than simply a waste metabolic product or a poisonous gas [27,38]. At physiological concentrations, CO has been documented to confer modulatory effects on vascular function, inflammation, apoptosis, proliferation, and many more through a multitude of signaling pathways [39,40]. Therefore, a new research field has emerged to explore the physiological properties and therapeutic benefits of CO rather than its toxicity. As exogenous CO would interact with the same biological targets as endogenous CO, the high affinity of CO for ferrous heme represents a barrier for the delivery of therapeutic CO to tissues. This implies that higher levels of exogenous CO that release supra-physiological amounts of CO are needed to mimic the effects of physiological concentrations of endogenous CO [39]. In experimental studies and clinical trials, COHb is a valuable tool to estimate the doses, the times, and the administrative modes of therapeutic CO that are safe for mammalian systems. Pharmacological usage of CO that may yield beneficial effects in animals has been observed with COHb levels not exceeding 15% [39]. Another study suggests that the maximal tolerable amounts of CO are in concomitance with about 10–12% COHb that is equivalent to the level of heavy smokers [41]. Clinically, inhalation of low-dose CO (100–125 ppm) for 2 h per day for four consecutive days led to a maximal individual COHb level of 4.5% in patients with stable chronic obstructive pulmonary disease. This remedy was found to be safe, feasible, and effective to reduce inflammatory responses [42].

**Fig. 1:**
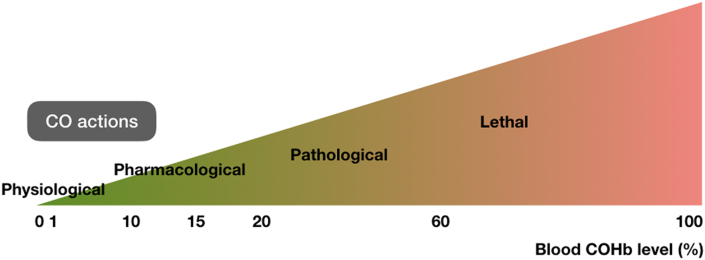
The dose-response of CO in mammalian systems. The measurement of blood COHb level is a useful tool to assess the actions of CO. Under physiological conditions, CO is endogenously produced at very low levels that lead to about 0.5–0.8% COHb in the blood. This gaseous molecule acts as an important signaling messenger that regulates multiple processes. For therapeutic purposes, the higher concentrations of CO are required. It has been suggested that pharmacological actions of CO are in concomitance with approximately 10–15% COHb. Moving upward, significant symptoms of CO poisoning are detectable at 20% COHb, and death may occur at the levels exceeding 60%.

To sum up, the fluctuation in CO levels would result in cellular interactions and signaling processes at varying degree. In general, proper concentrations of CO would play important role in the biology and medicine, while the overdose of CO would lead to the dysfunction of the cells and the dysregulation of signaling pathways which may eventually become pathological and even lethal (Fig. 1). “All substances are poisons; there is none that is not a poison. The right dose differentiates a poison from a remedy.”, as stated by Paracelsus. Therefore, the levels of CO are of the determinants to define the biological actions and pharmacological properties of this gas. This has been the subject of many reviews, some of which are referred here [[39], [40], [41]].

## 3 Short overview of carbon monoxide-releasing molecules (CO-RMs)

The novel concept of CO as a pivotal messenger molecule which plays multiple roles in the mammalian physiology has been increasingly accepted. The majority of endogenous CO comes from heme degradation which is enzymatically catalyzed by HO enzymes, with two major isoforms called inducible isozyme HO-1 and constitutive isozyme HO-2 [38]. A growing body of evidences has reported the protective effects of HO-1 in response to different stimuli through the interruption of various mechanisms of tissue injury, such as oxidative stress, inflammation, and cell cycle dysregulation, among others. Such protection of HO-1 may occur in almost all of tissues, and is mostly ascribed to its catabolic product namely CO [43]. Considering the role of HO-1/CO axis as a protective system in numerous experimental models of disease states [44], gaseous CO at low concentrations has gradually joined the armamentarium of preclinical and clinical therapeutics with potential applications in the treatment of many conditions [[18], [19], [20],[38], [39], [40], [41]].

A simple approach for the exogenous delivery of CO into the body is the inhalation of this gas. Due to its toxic nature, it is important to strictly control the delivery manner of CO to prevent both patients and healthcare workers from adverse health effects. In addition to the safety concern, this mode of administration remains other obstacles. A major drawback is the lack of tissue specificity. Upon the introduction into the body, there is no differentiation between healthy and pathological sites with respect to the distribution of gaseous CO through the circulation. Moreover, the relatively low water solubility of CO may further hamper its distribution to the target tissues, subsequently restricting its potential action at the target sites [20]. As a result, higher dosage of inhaled CO may be required to reach the sufficient concentration for its performance, yielding more difficulties regarding safe practice. To overcome these shortcomings, the research and development of pro-drugs capable of delivering CO in a steady and measurable fashion may provide a promising alternative to the direct administration of this therapeutic gas by inhalation. From this concept, Motterlini and colleagues, in 2002, the first time introduced the idea of utilizing transition metal carbonyl complexes as CO carriers that could mimic the action of HO-induced CO, serving as pro-drugs for CO delivery in a controlled manner, also termed CO-RMs [21]. Since then, a vast of metal carbonyl complexes has been extensively studied, presenting a major class of CO-RMs with promising feasibility and applicability for targeted distribution of therapeutic CO [45,46]. In addition to organometallic compounds, a number of other organic compounds have also been investigated in laboratory settings for their potential as CO-RMs [47,48].

**Fig. 2:**
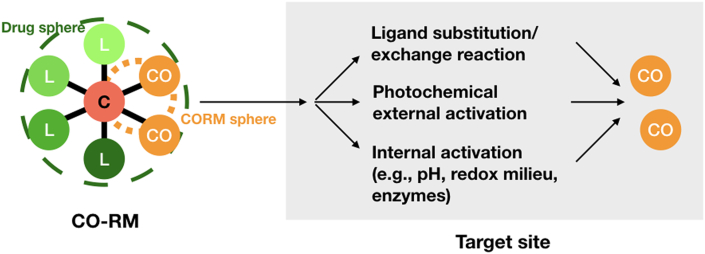
The two-dimensional schematic of the structure of CO-RMs and their modes of action of CO release. Surrounding the core, the CORM sphere (or coordination sphere) constitutes the inner part of CO-RM, and the drug sphere creates the outer. The CORM sphere determines the stoichiometry, the kinetics, and the trigger mechanism of CO release, while the drug sphere directs the pharmacological profile of CO-RMs. Upon their administration into the body, most CO-RMs induce the ligand substitution/exchange reaction with the medium to spontaneously liberate CO from the CORM sphere. Another potential mode of action is the photochemical external activation, resulting in the compounds so-called photoCO-RMs. Certain internal initiators such as pH, redox milieu, and enzymes have been exploited as trigger mechanisms; however, the study is still at preliminary stage. Note: C: core; CO: CO ligand; L: co-ligand.

The general concept of CO-RMs may refer to as substances that are administrated and distributed in the biological systems where they are activated to release CO in a controlled and stable fashion, eliciting biological activities in the target tissues and organs [21,49]. Structurally, CO-RMs typically consist of two major parts surrounding the core (e.g., transition metal centers in metal-based CO-RMs), that are, the CORM sphere and the drug sphere (Fig. 2). The CORM sphere, or coordination sphere, constitutes the inner part that is defined by the number and the spatial arrangement of CO ligands, determining the stoichiometry, the kinetics, and the trigger mechanism of CO liberation. Meanwhile, the drug sphere forming the outer part, that is defined by the periphery of co-ligands, modulates the partition ratio between body fluids and tissues, enabling tissue-specific targeting, thereby determining the pharmacological profile of CO-RMs [50]. Theoretically, different CO-RMs can be designed for a variety of particular biomedical applications by proper tuning CORM sphere or drug sphere or both to the desirable range.

A chief focus on CO-RMs is the mode of action by which CO moieties are released from the molecules, contributing to the site-specific delivery of exogenous CO. Several reviews of the representatives of CO-RMs with their specific trigger mechanisms have been well reported in literature [18,24,50,51]. For the early generations of CO-RMs, these CO donors can expose to high concentrations of biomolecules as potential ligands upon their administration into the body, yielding ligand substitution/exchange reactions with the medium to spontaneously release CO from the coordination sphere. This may lead to the drawback concerning the control of site-specific delivery because the tissue distribution of such CO-RMs mostly depends on the balance between their half-life in the specific medium with the necessary time to reach the target site [50]. To overcome this major shortcoming, a great effort has been put on the exploration of alternative modes of action through proper stimuli, where CO release is monitored. So far, one of promising strategies is the photochemical external activation so-called photoCORMs. The use of light with a broad range of wavelengths as an external trigger is believed to spatially and temporally deliver CO to the target sites in a tailored fashion [52]. Another possible mode is the application of thermal activation that in turn fosters ligand substitution/exchange reactions between CO-RMs and medium, leading to the increase in CO release [53]. In addition to external stimuli, several parameters in the cellular microenvironment, such as pH, redox milieu, and enzymes, have also been exploited as internal initiators. However, the study of internally triggered CO-RMs is still at tentative stage [50,51]. The characteristics of commercially available CO-RMs are summarized in Table 1.

**Table 1:** Characteristics of commercially available CO-RMs.

Name	Formula	Structure	Mode of action	Physicochemical properties	Ref
Dimanganese decacarbonyl, CORM-1	Mn_2_(CO)_10_		Photolysis	DMSO and water soluble; Concentration-dependent CO release by photodissociation	[21,24,54]
Tricarbonyl-dichloro-ruthenium (II) dimer, CORM-2	[Ru(CO)_3_Cl_2_]_2_		Solvent-ligand exchange	DMSO and lipid soluble; Average half-life of CO release is 1 min	[21,24]
Tricarbonylchloro (glycinato) ruthenium (II), CORM-3	RuCl(gly)(CO)_3_		Thermal degradation and solvent-ligand exchange	Water soluble; Average half-life of CO release is 1 min	[22,24]
Sodium boranocarbonate, CORM-A1	[H_3_BCO_2_]Na_2_		pH change	Water soluble; CO release in acidic milieu	[23,24]
Zerovalent molybdenum carbonyl anionic complex, ALF186	fac-[Mo(CO)_3_ (histidinato)]Na		Oxidation	Water soluble; Unstable under aerobic condition	[55]

Note: Reference [21] is the first publication reporting the identification, characterization, and pharmacological effects of a series of transition metal carbonyl complexes termed CO-RMs, notably CORM-1 and CORM-2. References [22,23] are those first introduced CORM-3 and CORM-A1, respectively.

The research field of CO-RMs as pro-drugs capable of delivering CO to cells and tissues has shown the potential which constitutes a promising CO-based therapeutic strategy. In fact, a multitude of experimental models has reported the beneficial effects of these molecules in various diseases [[21], [22], [23], [24], [25], [26]]. Importantly, it was observed that tricarbonylchloro(glycinato) ruthenium (II), known as CORM-3, can exhibit therapeutic effects without changing the COHb levels in vivo, suggesting high safety margin of CO pro-drugs for clinical applications [56]. However, the feasibility and applicability of CO-RMs as clinically effective therapeutics remain some challenges. Chemically, the structures of CO-RMs should be tuned for optimal physicochemical properties, ultimately yielding optimal efficacy. Pharmacologically, the release rates of CO-RMs should be controlled according to specific kinetics for proper pharmacodynamic and tissue distribution [57]. Another issue that poses a hurdle for the application of CO-RMs is the stigma of transition metal toxicity in these compounds. To date, a variety of metal complexes are being developed or employed as therapeutics in certain scenarios [58,59]. Therefore, it is improper to disqualify any compound from clinical development just as it contains transition metal. However, it is important to note that the addition of transition metals may increase the complexity of pharmaceutical development of an agent as compared to a conventional small molecule and protein/peptide-based drug [57]. Clearly, further research and development are highly warranted for the translation of CO-RMs into clinical stage in the future.

## 4 Anticancer activities of CO-RMs by targeting cancer hallmarks

It is recognized that the tightly-regulated growth of cells and tissues is disturbed in case of cancer. With this in mind, therapeutic agents targeting the mechanisms involved in the uncontrolled growth of cancer cells would become one of standards for cancer treatment. A potential approach is to control or terminate the rampant proliferation of cancer cells, for instance, through the induction of cell cycle arrest [60]. In addition, since cancer cells possess the characteristics that allow the abnormal survival beyond the normal life span, another promising strategy for cancer treatment is to eliminate cancer cells by inducing apoptosis which is well known a type of programmed cell death [61]. Interestingly, increasing body of literature has reported the potentials of CO-RMs as effective candidates that halt the abnormal behaviors of cancer cells via the inhibition of proliferation and/or execution of apoptosis in a panel of cancer models. So far, tricarbonyldichlororuthenium (II) dimer, also known as CORM-2, has been the most widely studied substance of CO-RMs in the context of cancer. CORM-2 has been demonstrated to yield anti-proliferative and pro-apoptotic effects on an array of cancer cell types, such as breast cancer [62], prostate cancer [63,64], colon and colorectal cancer [65], lung cancer [66], gastric cancer [67], pancreatic cancer [68], and lymphoma [69]. In line with in vitro experiments, different tumor-bearing rodent models have also indicated the inhibitory effects on tumor growth of this CO donor [63,65,[68], [69], [70], [71]]. Besides, a variety of novel CO-RMs have been designed and synthesized successfully, which have showed similar anticancer actions in both in vitro studies [[72], [73], [74], [75], [76], [77], [78], [79], [80]] and in vivo experiments [65,69,79]. In contrast, an in vitro study using human hepatocellular carcinoma cell lines showed that cell cycle arrest was attenuated by the pretreatment with CORM-2, interrupting the inhibition of cancer cell growth [81]. Similarly, CORM-2 was observed to increase the proportion of populations of cells retaining cancer stem cell properties in triple-negative breast cancer MDA-MB-231 cells and to stimulate the formation of mammospheres in these cancer cells [82]. Such contradictory findings suggest that CO-RMs may exhibit cell-specific effects on cell proliferation and cell death to determine the fate of cancer cells.

While blood vessels act as a complicated network that nurture body tissues, the process of new vessel formation called angiogenesis is a hallmark of cancer [83]. Such growth of vascular network is important to cancer progression because it not only enables cancer cells to acquire nutrients and oxygen but also to proliferate and metastasize to distant sites [84]. The significance of angiogenesis in cancer has led to the prospects that the regulation of this process would provide opportunities for cancer patients. Angiogenesis in cancer can be considered as a result of the imbalance between pro- and anti-angiogenic factors in favor of the former, leading to the disruption of dormant state [84]. In this sight, the maintenance of such equilibrium may offer benefits for the development of novel therapeutics of cancer. Notably, vascular endothelial growth factor (VEGF) is a key mediator of angiogenesis [85]. The production of VEGF and other factors by cancer cells can lead to the angiogenic switch, and the binding between VEGF with VEGF receptors (VEGFR1 and VEGFR2) which are expressed on vascular endothelial cells can result in angiogenesis [84,85]. Therefore, anticancer agents which target the VEGF/VEGFR axis has increasingly been a part of treatment in many cancer types [86]. A recent study using MDA-MB-231 breast cancer cell models found that the treatment with four commercially available CO-RMs, including CORM-1, CORM-2, CORM-3, and CORM-A1, significantly reduced the levels of excreted VEGF in these breast cancer cells, among which CORM-2 showed the highest efficacy followed by CORM-3 [87]. Based on their potency on VEGF reduction, CORM-2 and CORM-3 were further investigated for their ability to inhibit the activation of VEGFR2 in primary vascular endothelial cells upon VEGF stimulation. The results indicated that CORM-2 and CORM-3 could inhibit the phosphorylation of some downstream proteins of VEGFR2 signaling pathway, suggesting their capacity to disrupt the pro-angiogenic signal. In addition, the migration and tube formation of endothelial cells were also significantly attenuated by CORM-2 and CORM-3 [87]. Indeed, previous study also proposed the inhibitory effects of CORM-2 on VEGF-induced endothelial cell proliferation, migration, and capillary-like tube formation via the inhibition of VEGFR2 signaling pathway in human umbilical vein endothelial cells [88]. Thus, CORM-2 may be pursued as an agent for targeting the malignant angiogenesis in the settings of cancer. Likewise, a series of 15 new ruthenium-based CO-RMs was structurally modified from CORM-3 and showed in vitro anti-angiogenic behavior against MDA-MB-231 breast cancer cells. As compared to the lead compound, the novel complexes not only could reduce the upregulated VEGF expression from cancer cells as well as inhibit the activation of VEGFR2 and downstream proteins of vascular endothelial cells, but they could also suppress endothelial cell migration and new vessel formation [89]. The anti-angiogenic effects of CORM-2 have also been documented in a few other cancer models, such as in gastric cancer by mitigating IL-1β-induced IL-8 expression which is crucial for endothelial cell proliferation and angiogenesis [67], or in pancreatic cancer by inhibiting Akt phosphorylation which is important for cancer neovascularization [68]. Nevertheless, the effects of CO-RMs on angiogenesis in cancer remain highly controversial. The stimulatory effects of CO-RMs on angiogenesis has also been summarized in a recent review [26]. The opposite actions of CO-RMs on angiogenesis may be, in part, because of the specificity of related cells. Due to such perplexity, there is an urgent need to elucidate the role of CO-RMs in angiogenesis in the context of cancer.

One major hallmark of cancer is metastasis which involves the spread of cancer cells from the primary site to adjacent components and distant organs [90]. It is reported that metastasis is the leading cause of cancer morbidity and mortality, where it accounts for 90% of cancer deaths [91]. Therefore, a great concern is regarded to the prevention and treatment of cancer metastasis. It is acknowledged that the invasion and migration of cancer cells through the basement membrane is one of initial steps in the multi-step cascade of metastasis [90]. Interestingly, CORM-2 was found to efficiently inhibit the cell migration and invasion of non-small cell lung cancer Calu-3 cells [66]. Consistently, a recent study also reported that styrene-maleic acid copolymer-encapsulated CORM-2 could inhibit the cell migration and invasion of two colorectal cancer cell lines. Moreover, the administration with CORM-2 encapsulated formulation yielded a significant reduction of xenograft metastatic tumor growth in mice, which correlated to a lower number of metastasis loci [65]. Despite the paucity of information, these preliminary evidences have revealed the novel potential therapeutic application of CO-RMs in cancer, that is the inhibitory effect on the ability of cancer cells to invade and migrate to surrounding parts, thereby protecting body from metastasis.

To sum up, a wide range of cancer models has been subjected to CO treatment in form of CO-RMs, including breast cancer, prostate cancer, colon cancer, cervical cancer, gastric cancer, pancreatic cancer, skin cancer, lung cancer, and lymphoma. As summarized in Table 2, anti-proliferative, pro-apoptotic, and anti-angiogenic potentials are the major niches that have been documented while anti-metastatic effect has also been recently exploited. Although CO-RMs may behave as potential candidates that target some important hallmarks of cancers, still there is a lack of consistency in their efficacy. The conclusion until now is that the anticancer activities of CO-RMs are variable and cell-type specific. Clearly, it is mandatory to implement much more investigations to illuminate this issue.

**Table 2:** Summary of in vitro and in vivo studies regarding the anticancer activities of CO-RMs.

CO-RM(s)	Experimental cancer model	Results	Anticancer activities	Ref
Breast cancer				
CORM-2	Human invasive ductal breast carcinoma MCF-7 cells; Human triple negative breast cancer MDA-MB-231 cells	• Decreased the cell viability of both MCF-7 and MDA-MB-231 cells. • Attenuated heat shock protein 90 activity and its client proteins' expression, which involve in many hallmarks of cancer.	Inhibitory effects on the growth of breast cancer cells	62
fac-[MnBr(azpy)(CO)_3_]^(1)^; fac-[Mn(azpy)(CO)_3_(PPh_3_)](ClO_4_)^(1)^	MDA-MB-231 cells	• Designed photoCORMs exhibited the rapid CO release upon the exposure to low power visible light. • fac-[MnBr(azpy)(CO)_3_] reduced the cell viability of MDA-MB-231 cells through the photodelivery of CO under the control of visible light.	Induction of cell death of breast cancer cells through the photodelivery of CO	72
fac-[MnBr(CO)_3_(pbt)]^(2)^	MDA-MB-231 cells	• Designed photoCORM could cause approximately 50% reduction in the cell viability of MDA-MB-231 cells upon the illumination with broadband visible light.	Induction of cell death of breast cancer cells through the photodelivery of CO	73
fac-[Re(CO)_3_(pbt) (PPh_3_)](CF_3_SO_3_)^(2)^	MDA-MB-231 cells	• Designed photoCORM could eradicate 80% of MDA-MB-231 cells under the exposure to low-power UV light.	Induction of cell death of breast cancer cells through the photodelivery of CO	74
[Mn(CO)_3_(bpy)L]X^(3)^	MCF-7 cells	• Designed photoCORMs displayed considerable toxicity and inhibited the cell proliferation of MCF-7 cells upon UV light irradiation. • Different side groups showed different extent of anticancer effects.	Inhibition of cell proliferation of breast cancer cells through the photodelivery of CO	75
CORM-1; CORM-2; CORM-3; CORM-A1	MDA-MB-231 cells	• Reduced the VEGF levels in MDA-MB-231 cells. • Inhibited the phosphorylation of VEGFR2 and downstream proteins in primary vascular endothelial cells. • Decreased the migration and tube formation ability of endothelial cells.	Anti-angiogenic effect for targeting the malignant angiogenesis in breast cancer	87
CORM-3; A series of 15 ruthenium-based CO-RMs	MDA-MB-231 cells	• Reduced the upregulated VEGF expression in MDA-MB-231 cells. • Inhibited the activation of VEGFR2 and downstream proteins of vascular endothelial cells. • Inhibited the endothelial cell migration and new vessel formation.	Anti-angiogenic effect for treatment of angiogenesis in breast cancer	89
**Cervical cancer**				
fac-[MnBr(azpy)(CO)_3_]^(1)^; fac-[Mn(azpy)(CO)_3_(PPh_3_)](ClO_4_)^(1)^	Human cervical cancer HeLa cells	• Designed photoCORMs exhibited the rapid CO release upon the exposure to low power visible light. • fac-[MnBr(azpy)(CO)_3_] reduced the cell viability of HeLa cells through the photodelivery of CO under the control of visible light. • The morphological changes of HeLa cells upon CO exposure were typical of apoptosis.	Photo-initiated cytotoxicity and pro-apoptotic potential against cervical cancer cells	72
A series of Co_2_(CO)_6_HCC–CH_2_OCOR^(4)^	HeLa cells	• Designed CO-RMs showed structure-related cytotoxicity • Induced late apoptosis. • Arrested the cell cycle at G2/M phase.	Inhibitory effect on the growth of cervical cancer cells	76
**Prostate cancer**				
CORM-2	Prostate cancer (PCa) LNCaP and PC-3 cells	• Inhibited the viability and invasion of PCa cells. • Interrupted the energy metabolism of PCa cells. • Induced apoptosis of PCa cells.	Anticancer effects against prostate cancer in vitro and in vivo through the inhibition of cancer cell proliferation and invasion along with the induction of apoptosis	63
	Human PCa xenograft in nude mice	• Inhibited the tumor growth and induced apoptosis in PCa xenograft growth in nude mice.		
CORM-2/pluronic micelles	PC-3 cells	• CORM-2 was effectively encapsulated by pluronic, which released low levels of CO in the presence of cysteine. • Ultrasonic allowed CORM-2 micelles to release 4 times as much CO. • CORM-2 micelles decreased the cell viability of PC-3 cells up to 76% upon the ultrasonication.	CORM-2 micelles followed by ultrasonic activation can reduce the proliferation of prostate cancer cells	64
[FeII(CO)(N_4_Py)](ClO_4_)_2_	PC-3 cells	• Designed photoCORM exhibited the rapid CO release upon the irradiation with UV light, which showed potent cytotoxicity against PC-3 cells.	Growth inhibitory activity against prostate cancer cells	77
**Colon cancer and colorectal cancer**				
Styrene-maleic acid copolymer-encapsulated CORM-2	Murine colon carcinoma C26 cells; Human colon carcinoma SW480 cells	• Inhibited the cell proliferation, cell migration and cell invasion of both colorectal cancer cell lines.	Anti-proliferative and anti-metastatic effects in vitro and in vivo against colorectal cancer	65
	Murine colon carcinoma C26 tumor allograft in BALB/c mice	• Yielded the reduction of visible tumors, which correlated to a lower number of metastasis loci. • Decreased the ability to form subcutaneous tumors characterized by tumor volume and tumor growth.		
[Mn(CO)_3_(tpm)]PF_6_^(5)^	Human colorectal cancer HT29 cells	• Designed photoCORM released CO upon the irradiation with UV light, which showed cytotoxicity against HT29 cells	Photo-initiated cytotoxicity against colon cancer cells	78
fac-[RuII(CO)_3_Cl_2_L] with L = MBI or DMBI^(6)^; cis,*trans*-[RuII(CO)_2_Cl_2_(MBI)_2_]^(5)^	Human colon carcinoma SW480 cells;	• Designed complexes with MBI or DMBI ligands inhibited the cell growth of SW480 cells. • The bigger substituted benzimidazole ligands, the more considerable cytotoxicity.	[Ru(CO)x]-core complexes with benzimidazole ligands exhibit anticancer potential against colon cancer in vitro and in vivo	79
	Murine colon carcinoma CT-26 allograft in BALB/c mice	• Complex with MBI ligand yielded significant delay of tumor growth, whereas complex with DMBI ligand had no effect on tumor burden.		
Near infrared-mediated upconversion nanoparticle-based CO-RM	Human colorectal carcinoma HCT116 cells	• Designed photoCORM was taken up by HCT116 cells and showed dose-dependent cytotoxicity upon the irradiation. • Yielded significant reduction of the cell viability in other cancer cell lines, including murine melanoma B16 cells, murine mammary gland cancer 4T1 cells, MCF-7 cells, and HeLa cells, upon irradiation.	Photo-initiated cytotoxicity against various cancer cell lines	80
**Lung cancer**				
CORM-2	Human non-small cell lung cancer (NSCLC) Calu-3 cells	• Inhibited the cell proliferation, migration, and invasion of Calu-3 cells. • Promoted apoptosis of Calu-3 cells.	Anticancer effects against NSCLC in vitro through the inhibition of cancer cell proliferation, migration, and invasion along with the induction of apoptosis	66
CORM-2	Murine lung cancer CRL-1642 allograft in C57BL/6 mice	• Increased the body weight and thymus and spleen indices. • Histopathological analysis showed no evident cancer emboli in CORM-2 treated mice, which was accompanied with extensive fibrous hyperplasia, bleeding and necrosis. • Disrupted the signaling pathway associated to lung cancer	Anti-tumor activity against lung cancer in vivo	70
**Other cancers**				
CORM-2	Human gastric cancer AGS cells	• Inhibited IL-1β-induced IL-8 expression in AGS cells, which is known to induce the proliferation of endothelial cells that promotes angiogenesis. • Conditioned medium collected from IL-1β-exposed AGS cells promoted the in vitro growth of endothelial cells, whereas CORM-2 or IL-8 neutralizing antibody could abolish the proliferation-stimulatory effect of the medium.	Inhibitory effect on the cell proliferation of endothelial cells and angiogenic effect of IL-8 caused by IL-1β-stimulated AGS cells	67
CORM-2	Pancreatic cancer CAPAN-2, BxPc3, and PaTu-8902 cells	• Inhibited the cell proliferation of three pancreatic cancer cell lines. • Inhibited the Akt phosphorylation, which is important for cancer neovascularization, in CAPAN-2 and PaTu-8902 cells.	Anti-proliferative and anti-angiogenic effects in vitro and in vivo against pancreatic cancer	68
	CAPAN-2 xenograft in athymic mice	• Increased the survival rate. • Affected de no angiogenesis characterized by decrease of CD31-positive vessels.		
CORM-2; Folic acid-tagged nanoemulsions loaded CORM-2	B-cell lymphoma A20 cells	• Either the incorporated nanoemulsions or CORM-2 reduced the cell viability of A20 cells, where the former yielded stronger effect than the latter.	Folic acid-tagged nanoemulsions could specifically deliver CORM-2 to cancer cells expressing folate receptors on the cell surface, enhancing anticancer action	69
	A20 lymphoma tumor xenograft in BALB/c mice	• Either the incorporated nanoemulsions or CORM-2 enhanced the survival of mice and inhibited the tumor growth, where the former yielded stronger effects than the latter.		
CORM-2	Photocarcinogenesis induction in female inbred albino Skh:hr-1 hairless mice	• Reduced the inflammatory erythema and reduced the average tumor multiplicity. • Produced the moderate inhibition of early tumor appearance, increased the regression of established tumors, and inhibited the development of malignant and locally invasive large tumors in dose-dependent manner	In vivo anti-photocarcinogenic action through the delivery of CO, providing protection from skin cancer	71

^(1)^ azpy = 2-phenylazopyridine. ^(2)^ pbt = 2-(2-pyridyl)benzothiazole. ^(3)^ bpy = 2,2′-bipyridine; X = hexafluorophosphate, trifluoromethanesulfonate; L = imidazole, methylimidazole, benzimidazole, *N*-benzylbenzimidazole, *N*-(4-chlorobenzyl)benzimidazole. ^(4)^ R = 2- acetyloxybenzoic acid, 2-hydroxy-benzoic acid, 3-phenyl-2-propenoic acid, ibuprofen, naproxen, glycyrrhetinic acid, glycyrrhizic acid. ^(5)^ tpm = tris(pyrazolyl)methane. ^(6)^ MBI = *N*-methylbenzimidazole; DMBI = 5,6-dimethylbenzimidazole.

## 5 Mechanisms behind the anticancer activities of CO-RMs: an outlook from ROS biology and medicine

**Fig. 3:**
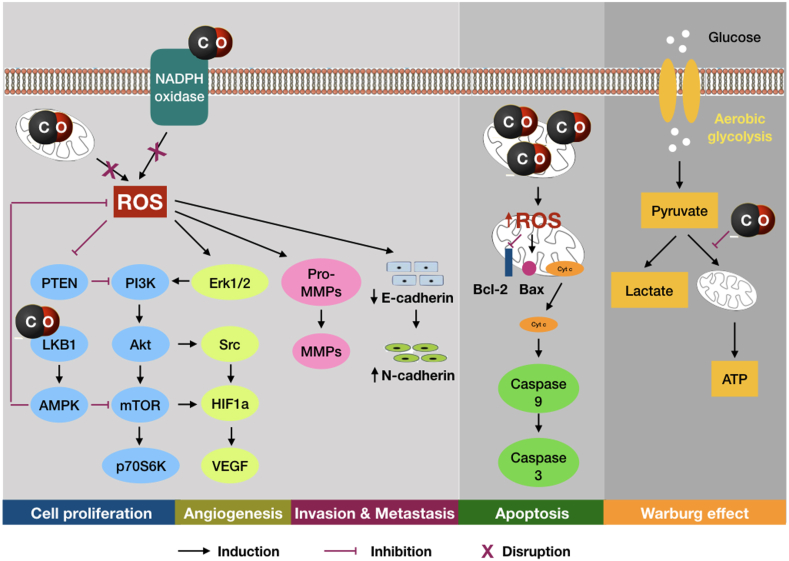
Proposed mechanisms behind the anticancer activities of CO released from CO-RMs. The anticancer actions of CO-RMs is mainly associated with the inhibition of cell proliferation, angiogenesis, and invasion and metastasis as well as the induction of apoptosis. As mitochondria and NADPH oxidase, which are known the major sources of intracellular ROS, have been implicated as the targets of CO action, ROS seem likely to play important roles in the signaling pathways by which CO-RMs exert their actions. While CO may downregulate the ROS generation to inhibit different pro-tumorigenic pathways, CO at higher concentrations can increase the levels of ROS to induce the mitochondrial-mediated apoptotic pathway. Thus, CO-RMs can take advantages of both sides of ROS to provide therapeutic benefits in specific scenario. Moreover, CO-RMs may also act as promising candidates that target the cancer metabolic reprogramming by eliciting anti-Warburg effect.

In aerobic organisms, the metabolism of molecular oxygen inevitably generates an array of oxygen-containing reactive species termed reactive oxygen species (ROS) which play pivotal roles in both physiology and pathophysiology [92]. Under physiological conditions, ROS are maintained in a dynamic balance by the antioxidant defense system to act as a pleiotropic signaling that drive different regulatory pathways [93]. During the course of cancer, the progressive disturbance of redox equilibrium so-called oxidative stress can be resulted from the overproduction of ROS in relation to the antioxidant capacity, contributing to the induction of cancer hallmarks and enabling characteristics [6,93]. Although cancer cells typically exhibit abnormal redox homeostasis [94], “*the dose makes the poison*” is a noteworthy viewpoint [95]. Pro-tumorigenic pathways are accompanied by higher levels of ROS, but anti-tumorigenic pathways may occur under excessive oxidative burden, suggesting the dual role of ROS in cancer [96]. This section suggests the sophisticated mechanisms underlying the anticancer activities of CO-RMs through the regulation of ROS (Fig. 3).

### 5.1 Inhibition of pro-tumorigenic pathways through the downregulation of ROS

**Downregulating the ROS generation.** Mitochondria, the major source of intracellular ROS, have been reported as an important target of CO action because certain mitochondrial cytochromes responsible for the respiration possess heme functional groups that serve as binding sites for CO [33,97]. Thus, the administration of CO can impair the mitochondrial function and respiration, thereby downregulating the increased ROS production [98]. In addition, the family of NADPH oxidases are cytochrome-containing enzyme complexes responsible for the generation of a species of ROS so-called superoxide anion. As a result, NADPH oxidases are also recognized as a target of CO action, contributing to the inhibition of ROS production [97]. Recently, the effect of CORM-2 on free radicals per se and hydrogen peroxide-induced oxidative stress was investigated in peripheral blood mononuclear cells and human promyelocytic leukemia HL-60 cells. The results indicated the potent antioxidant properties of CORM-2 in both cell lines, but more pronounced in HL-60 cells, as evidenced by the markedly reduction of ROS levels [99]. The downregulation of ROS may represent a mechanism by which CO-RMs exert inhibitory effects on the relevant pro-tumorigenic signaling pathways.

**Targeting the self-sufficiency in proliferation signals.** One of fundamental hallmarks of cancer involves the ability of cancer cells to sustain their cell proliferation [100]. The phosphatidylinositol 3-kinase/protein kinase-B/mammalian target of rapamycin (PI3K/Akt/mTOR) signaling cascade is one of crucial pathways which is aberrantly activated in many cancers, resulting in certain disturbances in the regulation of cell proliferation and various cellular processes [101]. The administration of CO using CORM-2 was found to inhibit the abnormal cell proliferation and malignant growth in orthotopic allograft lung tumor mice models by disrupting the PI3K/Akt/mTOR pathway, as demonstrated by the reduction of PI3K, Akt, and mTOR phosphorylation. The downstream effector of mTOR namely p70 S6 kinase, which promotes the sustained cell growth and proliferation, was downregulated by CORM-2 as well [70]. Other studies on colorectal cancer or pancreatic cancer also observed the reduction of Akt phosphorylation following CORM-2 treatment [65,68]. These preliminary evidences suggest the ability of CO-RMs to compromise the self-sufficiency of cancer cells in proliferative signals by inhibiting PI3K/Akt/mTOR pathway. Intriguingly, recent studies have highlighted the interplay between redox stress and PI3K/Akt signaling in cancer progression. PI3K/Akt signaling may positively regulate ROS generation in cancer cells through the modulation of mitochondrial bioenergetics and the activation of NADPH oxidases [102], whereas high levels of ROS may activate PI3K/Akt signaling in several ways [103]. Therefore, it is possible to propose a circuitry underlying the anticancer activities of CO-RMs, in which the inhibition of ROS generation may result in the deactivation of PI3K/Akt/mTOR pathway, and vice versa.

**Targeting the insensitivity to antiproliferation signals**. Another trait of cancer involves the ability of cancer cells to evade the programs for negative regulation of cell proliferation which mainly depend on the actions of tumor suppressors [100]. Liver kinase B1 (LKB1) is a tumor suppressor that regulates various cellular processes through the activation of 5′-adenosine monophosphate-activated protein kinase (AMPK), and the dysregulation of LKB1/AMPK signaling has been implicated in many cancers. Moreover, AMPK may act as an inhibitor of cell growth by inhibiting mTOR pathway [104]. Importantly, literature has also reported the involvement of LKB1/AMPK pathway in the maintenance of redox homeostasis by alleviating ROS generation and promoting ROS scavenging [104]. The LKB1/AMPK signaling pathway was demonstrated as an underlying mechanism by which CO elicit anticancer effects. The treatment with CORM-2 led to the increase of LKB1 expression and AMPK phosphorylation as well as the decrease of mTOR activation in prostate cancer cells and tumor xenograft mice. Importantly, the knockdown of LKB1 significantly reduced the effect of CORM-2 on the proliferation of cancer cells as well as the phosphorylation of AMPK and mTOR [63].

**Targeting the angiogenesis.** Angiogenesis, or tumor-associated neovasculature, addresses the needs of tumors in terms of sustenance and metabolism, promoting neoplastic growth [100]. An increasing body of evidences has reported the involvement of high ROS concentrations in the induction of angiogenesis. The signaling cascade by which ROS mediate angiogenesis mainly involves the expression of VEGF in several ways. The major pathway is the complex network of PI3K/Akt/mTOR, p70 S6 kinase, phosphatase and tension homolog (PTEN), and mitogen-activated protein kinases (MAPKs) signals via hypoxia-induced factor 1-alpha [105,106]. It has also reported that ROS may lead to the activation of endothelial cells and the induction of angiogenesis through extracellular signal-regulated kinase (Erk)/PI3K/Akt/proto-oncogene tyrosine-protein kinase (Src) pathway [106]. In addition to the hypoxia-dependent cascade, ROS can induce angiogenesis through the oxidative lipid ligands which activate the nuclear factor kappa subunit B (NF-κB) transcription factor via Toll-like receptors (TLRs) [105,106]. As presented above, CO-RMs may exert anti-angiogenic effects by targeting the VEGF/VEGFR axis. Mechanistically, such effects may associate to the inhibition of Akt phosphorylation as observed in CORM-2-treated colorectal cancer cells [65]. By using breast cancer cell models, anti-angiogenic effects of CO donors may also be due to the significant inhibition of Erk1/2 phosphorylation, and in a lesser extent, the Src phosphorylation [87]. In addition, a few studies on different cancer models have reported the inhibitory effects of CORM-2 on the levels of p70 S6 kinase, NF-κB, and TLR4, suggesting the disruption of hypoxia-independent angiogenesis [70,107].

**Targeting the invasion and metastasis.** Matrix metalloproteinases (MMPs) are essential enzymes capable of degrading extracellular matrix which pave the way for cancer cells to migrate and invade surrounding components [108]. In fact, the inhibition of MMP-2 and MMP-9 was suggested to be responsible for the anti-invasive and anti-metastatic effects of CORM-2 in colorectal cancer cells [65]. However, the upstream regulatory pathways remain unknown. Literature has reported the role of ROS in the migration and invasion of cancer cells that are initial steps for metastasis [105,106]. It has also been documented that the invasive abilities of cancer cells may associate with the increase of MMP-9 activities in ROS-dependent manner [109]. By counteracting ROS overproduction, CO-RMs seem able to inhibit the activation of MMPs, preventing the migration and invasion of cancer cells. On the other hand, epithelial to mesenchymal transition (EMT) is another major step of metastasis which may be triggered by the high levels of ROS [105,106]. EMT involves the loss of epithelial markers (e.g. E-cadherin), the induction of mesenchymal markers (e.g., *N*-cadherin), and the upregulation of transcription factors (e.g., Snail) [110]. Lesser invasion was observed in prostate cancer cells treated with CORM-2, suggesting its anti-metastatic effects. Moreover, the levels of *N*-cadherin and SNAI2 were significantly decreased while the levels of E-cadherin were markedly increased upon CORM-2 treatment. Similar results were observed in tumor xenograft mice, further indicating the inhibitory effects of CORM-2 on EMT [63].

**Intervening the enabling characteristics.** The acquisition of cancer hallmarks requires two enabling characteristics. One enabling characteristic is genome instability which generates a diversity of genetic mutations and changes that orchestrate hallmark capabilities, and another one is inflammation which promotes hallmark functions and tumor progression through various means [100]. Some studies on cancer models have observed the inhibitory effects of CO-RMs on these enabling characteristics. For instance, CORM-2 was found to reduce hydrogen peroxide-induced DNA oxidative damage in human promyelocytic leukemia cells [99]. In addition, tumor-promoting inflammation was also inhibited by CORM-2, as demonstrated by the reduction of serous levels of inflammatory markers (i.e., tumor necrosis factor alpha, IL-1β, and IL-6) in orthotopic lung cancer mice [70]. Intriguingly, growing body of literature has reported the involvement of ROS in the development of both genomic instability and inflammation [5,9,105]. Because CO-RMs have been documented as potent candidates in a variety of diseases owing to their broad activities, particularly anti-inflammation and antioxidant [20,25], it is rationale to believe that ROS may be an important signaling by which CO-RMs regulate two enabling characteristics of cancers. Further studies are required to elucidate this assumption.

### 5.2 Induction of apoptosis through the upregulation of ROS

Apoptosis is a type of programmed cell death typically executed by specialized proteases termed caspases [111]. One of two major pathways for the induction of apoptosis depends on mitochondrial redox state [112]. This pathway is initiated by the mitochondrial outer membrane permeabilization (MOMP) which is controlled through the interactions among proteins of Bcl-2 family. In particular, pro-apoptotic proteins such as Bax promote MOMP while anti-apoptotic proteins such as Bcl-2 inhibit MOMP. Following MOMP, the release of apoptogenic factors, such as cytochrome *c*, from mitochondria into the cytoplasm results in the activation of caspase-9 which in turn activates effector caspases such as caspase-3 [113]. Interestingly, the levels of active (cleaved) caspase-3, -8, -9 were increased in prostate cancer cells treated with CORM-2 [63]. Moreover, CORM-2 treatment also significantly reduced the ratio of Bcl-2/Bax while increased the expression of caspase-3 and cytochrome *c* in non-small cell lung cancer cells [66]. These evidences suggest that CORM-2 may yield Bcl-2/Bax-dependent MOMP induction and cytochrome c-dependent caspase activation, leading to mitochondria-dependent apoptosis where ROS seem to be crucial regulatory signaling. In fact, mitochondria have been implicated as a major target of CO action [33,97]. It is noteworthy that the effects of CO on mitochondrial function may vary depending on CO concentrations, duration of exposure, and cell types [114]. In particular, CO at higher concentrations can inhibit the mitochondrial respiration, upregulate ROS generation, and induce oxidative stress [[115], [116], [117]], which in turn may activate apoptotic pathway.

### 5.3 Induction of anti-Warburg effect

It has been proposed that ROS homeostasis and cellular metabolism represent a critical liaison in cancer. Thus, the research and development of cancer therapy should include the identification and regulation of essential nodes associated to cancer metabolism that can be driven by the changes in redox status [118]. The Warburg effect, or aerobic glycolysis, describes the increased conversion of glucose to pyruvate followed by the synthesis of lactate in the presence of oxygen [119], while the normal glycolysis relies on mitochondrial oxidative phosphorylation (OXPHOs) [120]. Switching to aerobic glycolysis is a manner of metabolic reprogramming which is a hallmark of cancer [100]. Exogenous CO has been reported to target the mitochondrial activities in prostate cancer cells, as evidenced by higher oxygen consumption, free radical generation, and mitochondrial collapse, suggesting the ability of CO to induce higher rates of OXPHOs [121]. The effect of CO released from CORM-2 on the glycolysis levels was also documented in breast cancer cells, where CORM-2 yielded a significant reduction in the glycolysis change rate [87]. However, the precise mitochondrial changes that underline the anti-Warburg effect of CO-RMs remain unknown. Literature has proposed that mitochondrial uncoupling, a process in which electron transport chain is not employed to drive the ATP synthesis and other functions, can mediate the metabolic shift to aerobic glycolysis in certain cells [122,123]. Intriguingly, it was found that CO released from CORM-401 can induce the uncoupling of mitochondrial respiration, and this increased respiration was associated with the inhibition of glycolysis in endothelial cells [124]. These results suggest the importance of CO liberated from CO-RMs in the metabolic reprogramming of the endothelium which may be implicated in the pathological angiogenesis. Similarly, earlier work also reported that low concentrations of CORM-3 can act as mitochondrial uncoupling factor [125]. Although studied experimental models are non-cancerous, they have provided important clues for disclosing CO mechanisms associated to mitochondrial changes. It can be implicit that exposure to CO can take advantage of the Warburg physiology to compel cancer cells to consume more oxygen, in turn, affect the metabolic reprogramming. This would ultimately lead to growth inhibition, exhaustion, and death of cancer cells. Altogether, in addition to their ability to regulate ROS generation, available evidences suggest the feasibility of CO-RMs as candidates that target the cancer metabolism, thereby improving the outcome of cancer treatment.

## 6 Conclusion and future prospects

CO is an attractive agent for its availability, cost-effectiveness, and easy-to-perform advantages. This may open up a therapeutic window in cancer by tailored and controlled delivery of CO to the site of interest at a particular time via CO-RMs. Mechanistically, there is a plausible complicated network of signaling pathways underpinning the anticancer activities of CO-RMs, in which ROS may lie in the center. However, the dual nature of ROS in cancer remains a conundrum, where the up- or down-regulation of ROS generation can have different impact on cancer initiation and progression. It is thus necessary to implement more investigations for the exploitation of ROS in cancer therapy. Furthermore, the insufficiency of evidences demonstrating the direct relationship between ROS and other signaling molecules in response to CO derived from CO-RMs in cancer models constitutes another limitation. The application of genetic and/or pharmacological approaches that intervene ROS and relevant signals in cancer models treated with CO-RMs may bridge this gap, providing a more panoramic and precise visualization about the underlying molecular mechanisms. Finally, although some of available CO-RMs have been confirmed their beneficial effects in various models of cancer both in vitro and in vivo, the feasibility and applicability of CO-RMs as clinically effective therapeutics remain several hurdles. This brings the task of developing the pharmaceutical CO-RMs equipped with the safety and profiles required for clinical use.

## Declaration of competing interest

The authors report no conflicts of interest in this work.
